# Basket trial of TRK inhibitors demonstrates efficacy in TRK fusion-positive cancers

**DOI:** 10.1186/s13045-018-0622-4

**Published:** 2018-06-07

**Authors:** Yu Chen, Ping Chi

**Affiliations:** 1 Human Oncology and Pathogenesis Program, Memorial Sloan Kettering Cancer Center, New York, NY 10065 USA; 2 Department of Medicine, Memorial Sloan Kettering Cancer Center, New York, USA; 3 Department of Medicine, Weill Cornell Medical College, New York, USA

## Abstract

Unlike many conventional cancers with preferential patterns of oncogenic genetic alterations, TRK fusions resulting from NTRK1/2/3 genetic alterations drive oncogenic transformations in more than 20 different malignancies over diverse tissue/cell lineages, in both children and adults. A recent “basket” study of larotrectinib, a TRK inhibitor, has demonstrated significant efficacy in TRK fusion-positive tumors of all types from infants to the elderly. Here, we discuss the larotrectinib study and perspectives and challenges in developing “tumor-agnostic” targeted therapies in rare tumors.

## Background

Traditionally, cancers are classified and treated based on their pathologic classification and tissue of origin. Advances in sequencing technology and large-scale cancer genomics effort (e.g., the International Cancer Genome Consortium and The Cancer Genome Atlas (TCGA) have identified many targetable driver genetic alterations across different tumor types and have shifted the cancer sub-classification based on driver genetic alterations. For example, lung adenocarcinomas are further sub-classified by *KRAS* and *EGFR* mutations and *ALK* and *ROS1* translocation. Most “driver” genetic alterations are preferentially found in certain cell/tissue lineages. For example, the *BRAF*^V600^ mutation occurs at high frequencies in specific tumor types, e.g., melanoma, thyroid cancer, hairy cell leukemia, Langerhans cell histiocytosis, and colorectal cancer, and at significantly lower frequencies in other tissue lineages. The responses to *BRAF*^V600^-targeted therapies are not uniform, with some cancer types (e.g., colorectal cancer) exhibit tissue lineage-specific primary resistance [1–3], underlining the importance of the tissue lineage-specific cellular context.

## TRK fusions are rare but drive oncogenesis in diverse tissue lineages

The *NTRK1*, *NTRK2*, and *NTRK3* genes, encoding the tropomyosin receptor kinases (TRK), TRKA, TRKB, and TRKC, respectively, are receptor tyrosine kinases that are normally expressed in the nervous system [4]. Physiologically, TRK receptor tyrosine kinases are activated by binding of mature neurotrophins, which mediate neuronal survival and synaptic plasticity in the central nervous system [4]. NTRK genetic alterations (e.g., translocations) resulting in TRK fusion proteins can lead to ligand-independent activation of TRK kinases and drive oncogenic transformation [5–7]. To date, TRK fusions are found in more than 20 different tumor types. With the exception of several rare tumor types (e.g., secretory breast carcinoma, mammary analog secretory carcinoma, congenital fibrosarcomas, and congenital mesoblastic nephroma), the majority of the TRK fusions occur in low frequencies in a variety of common cancers over a diverse tissue/cell lineages (e.g., lung adenocarcinoma, sarcoma, acute myeloid leukemia, colorectal cancer) [6, 7] (Table 1). The rarity of TRK fusions and the heterogeneity of tumor types present incredible challenges to clinically evaluate TRK inhibitors. Diagnostically, because of large introns, these fusions are difficult to detect using multiplex targeted exome capture panels (e.g., FoundationOne®, MSK-IMPACT™).

**Table 1:** Rare TRK fusions in diverse tumor types

Tumor type	NTRK1/2/3 involved (frequency)	Detection methods
Ph-like acute lymphoblastic leukemia	NTRK3 (1/154)	RNA-seq, whole-genome seq, whole-exome seq [18]
Appendiceal adenocarcinoma	NTRK (unspecified) (2/97)	MALDI-TOF mass spectroscopy genotyping (Sequenom), targeted NGS (MSK-IMPACT) [19]
Astrocytoma	NTRK2 (3/96)	RNA-seq, whole-genome seq [20]
Breast invasive carcinoma	NTRK3 (1/1072)	RNA-seq (TCGA) [21]
Intrahepatic cholangiocarcinoma	NTRK1 (1/28)	Targeted NGS [22]
Colon adenocarcinoma	NTRK1 (8/1559)	IHC, RT-PCR [23]
	NTRK1 (1/66)	IHC, RT-PCR [24]
	NTRK3 (2/286)	RNA-seq (TCGA) [21]
GIST	NTRK3 (1/186)	Targeted NGS (Foundation Medicine) [25]
	NTRK3 (1/31)	RNA-seq, FISH, RT-PCR [26]
Glioblastoma	NTRK1 (1/157)	RNA-seq (TCGA) [21]
	NTRK1 (2/185)	RNA-seq (TCGA and other) [27]
	NTRK1 (3/115)	Targeted NGS [28]
Brain low-grade glioma	NTRK2 (2/461)	RNA-seq (TCGA) [21]
Pediatric DIPG and non-brainstem high-grade glioma	NTRK1 (3/127)	RNA-seq, whole-genome seq [29]
	NTRK2 (3/127)	RNA-seq, whole-genome seq [29]
	NTRK3 (2/127)	RNA-seq, whole-genome seq [29]
Head and neck squamous cell carcinoma	NTRK2 (1/411)	RNA-seq (TCGA) [21]
	NTRK3 (1/411)	RNA-seq (TCGA) [21]
Congenital mesoblastic nephroma	NTRK3 (5/6)	RT-PCR, FISH [30]
	NTRK3 (10/15)	RT-PCR [31]
	NTRK3 (13/19)	FISH [32]
Infantile (congenital) fibrosarcoma	NTRK3 (10/11)	RT-PCR, IHC [33]
	NTRK3 (5/5)	RT-PCR, FISH [30]
Lung adenocarcinoma	NTRK1 (3/91)	Targeted NGS (Foundation Medicine), FISH [34]
	NTRK2 (1/513)	RNA-seq (TCGA) [21]
Breast secretory carcinoma	NTRK3 (12/13)	RT-PCR, FISH [35]
	NTRK3 (9/9)	FISH, targeted NGS [36]
Mammary analogue secretory carcinoma (MASC) of salivary glands	NTRK3 (13/14)	RT-PCR, FISH [37]
	NTRK3 (15/15)	FISH [38]
	NTRK3 (5/6)	FISH, targeted NGS [36]
	NTRK3 (16/20)	RT-PCR, FISH [39]
Melanoma (skin cutaneous)	NTRK3 (1/374)	RNA-seq (TCGA) [21]
Spitz tumors and spitzoid melanoma	NTRK1(23/140)	Targeted NGS(Foundation Medicine), FISH, IHC [40]
Sarcoma (NOS)	NTRK1 (1/103)	RNA-seq (TCGA) [21]
Uterine sarcoma	NTRK1 (3/97)	RNA-seq, FISH, IHC, targeted NGS [41]
	NTRK3 (1/97)	RNA-seq, FISH, IHC, targeted NGS [41]
Thyroid carcinoma	NTRK1 (5/498)	RNA-seq (TCGA) [21]
	NTRK3 (7/498)	RNA-seq (TCGA) [21]
Papillary thyroid carcinoma	NTRK1 (15/119)	RT-PCR [42]
	NTRK1 (2/38)	Southern [43]
	NTRK1(pediatric) (1/27)	Targeted NGS [44]
	NTRK3(radiation-associated) (2/26)	RNA-seq [45]
	NTRK3(radiation-associated) (9/62)	RNA-seq [46]
	NTRK3(sporadic) (3/151)	RNA-seq [46]
	NTRK3(pediatric) (6/27)	Targeted NGS [44]

## TRK inhibitor larotrectinib demonstrates efficacy in a basket trial

Recently, Drilon and colleagues reported a phase I/II clinical trial to evaluate the safety and efficacy of larotrectinib, a highly selective small-molecule inhibitor of all three TRK proteins, using a novel “basket” trial design that enrolled patients based on NTRK genetic alterations regardless of age or tumor types [8]. A total of 55 patients (ages 4 months–76 years old) with 16 different tumor histologies were treated on three protocols, and the results were pooled. The investigators found that larotrectinib was generally well-tolerated with < 5% treatment-related grade 3 or 4 adverse events. The overall RECIST response rate was 75% (95% confidence interval, 61–85) by independent review and 80% (95% confidence interval, 67–90) by investigator assessment. At 1 year, 71% of patients are with ongoing responses and 55% of patients remain progression-free. The median duration of response and progression-free survival has not been reached after 8.3 and 9.9 months of median follow-up, respectively. Importantly, responses were observed in nearly all tumor types and age groups. Three of the six patients who did not response to larotrectinib (primary resistance) had undetectable TRK proteins by immunohistochemistry (IHC) despite molecularly identified TRK fusion at a screening in local laboratories. In the ten patients who progressed after an initial response for at least 6 months, nine had identifiable secondary resistant mutations in *NTRK1* or *NTRK3*, including substitutions in the solvent front position (*NTRK1* G595R or *NTRK3* G623R), gatekeeper mutation (*NRTK1* F589 L), and the xDFG position *NTRK1* G667S or *NTRK3* G696A). The acquired resistance mechanisms have been described for other oncogenic kinase-targeted therapies [9–11]. The next generation of TRK inhibitors is in development to overcome the acquired resistance in TRK [7, 12] (see Table 2).

**Table 2:** TRK inhibitors currently in clinical development

Drug name	Targets	Development stage	Clinical trial identifier	Company
LOXO-101 (larotrectinib)	NTRK1/2/3	Phase II	NCT02122913 NCT02637687 NCT02576431 NCT03213704	Loxo Oncology
LOXO-195	NTRK1/2/3 (resistant)	Phase I/II	NCT03215511	Loxo Oncology
RXDX-101 (entrectinib)	NTRK1/2/3, ALK, ROS1	Phase I/II, Phase II, Phase I/Ib	NCT02097810 NCT02568267 NCT02650401	Ignyta
TPX-0005 (ropotrectinib)	NTRK1/2/3, ALK, ROS1 (resistant), JAK2, SRC, DDR1, FAK	Phase I/II	NCT03093116	TP Therapeutics
LY2801653 (merestinib)	NTRK1/2/3, MET, MST1R, FLT3, AXL, MERTK, TEK, ROS1, DDR1/2, MKNK1/2	Phase II	NCT02920996	Eli Lilly and Company
DS-6051b	NTRK1/2/3, ROS1	Phase I	NCT02675491 NCT02279433	Daiichi Sankyo
PLX7486	NTRK1/2/3, CSF1R	Phase I	NCT01804530	Plexxikon/Daiichi Sankyo
MGCD516 (sitravatinib)	NTRK1/2/3, MET, KIT, PDGFRA, KDR, DDR2, RET, CBL	Phase I/Ib	NCT02219711	Mirati Therapeutics

## Future perspectives

The study by Drillon et al. [8] comes on the heels of several basket trials, including the AKT inhibitor AZD5363 in *AKT1* E17K-mutant tumors [13], the PD1 inhibitor pembrolizumab in mismatch repair deficient tumors [14], and the pan-HER kinase inhibitor neratinib in HER2- and HER3-mutant tumors [15], with variable clinical success. This current study provides a compelling case for tumor-agnostic, molecular-driven “basket” approaches for clinical investigations of rare driver mutations across diverse tumor types. It paves a clinical pathway to effective therapeutics for patients with rare tumors and rare driver mutations. In addition to larotrectinib, there is a variety of TRK inhibitors currently in clinical development (Table 1), including next-generation TRK inhibitors that can overcome acquired resistance (e.g., LOXO-195, TPX-0005).

Despite the early clinical success with new generations of TRK inhibitors and novel trial design, the challenges remain for real-time identification of rare TRK fusions. What would be the ideal diagnostics methodology? DNA-based next-generation sequencing (NGS) assays have relatively high false-negative and false-positive rate and do not identify novel fusions. RNA-based NGS assays (e.g., Archer Dx) can detect novel fusions and has reasonable sensitivity. However, both DNA- and RNA-based NGS assays can be costly and effort intensive. Alternatively, IHC of TRK is a sensitive and efficient method for identification of TRK expression [16, 17]. Nevertheless, it would not readily discriminate TRK fusion arising through genetic alterations where TRK inhibitors can be highly effective from full-length TRK expression in tumors inherited through development where the functional significance of TRK expression and clinically impact is unknown. TRK IHC can also be associated with false positives in certain tissue and tumor types. Furthermore, unlike NGS-based assays, IHC cannot be easily multiplexed into a panel without added cost and effort. While TRK IHC can be easily justified for high-prevalence tumors (e.g., congenital fibrosarcoma or secretory breast carcinoma), its role in low-prevalence common tumors such as colorectal cancer becomes more debatable. Importantly, it is unclear which diagnostic modality, NGS of NTRK alterations or IHC of TRK expression, is more predictive of response to TRK inhibitors. In the NEJM by Drilon et al., three out of the six non-responders to larotrectinib did not have centrally confirmed TRK expression by pan-TRK IHC, despite the detection of NTRK rearrangement by NGS in the local laboratory [8]. This observation suggests that TRK expression by IHC may be necessary for response. Currently, Pan-TRK IHC and Illumina NGS (RNA and DNA assays in one design) are both being developed as companion diagnostics to larotrectinib and other TRK inhibitors.

With a shifting paradigm of identifying genetic alterations in a tumor-agnostic manner, the development of a single assay that can identify multiple types of actionable genetic alterations would be paramount. In the meantime, TRK IHC would be a reasonable initial diagnostics for rare tumors where TRK fusions are frequent, and possibly common tumors where driver mutations are absent.
